# Brain Activity during Mental Imagery of Gait Versus Gait-Like Plantar Stimulation: A Novel Combined Functional MRI Paradigm to Better Understand Cerebral Gait Control

**DOI:** 10.3389/fnhum.2017.00106

**Published:** 2017-03-06

**Authors:** Matthieu Labriffe, Cédric Annweiler, Liubov E. Amirova, Guillemette Gauquelin-Koch, Aram Ter Minassian, Louis-Marie Leiber, Olivier Beauchet, Marc-Antoine Custaud, Mickaël Dinomais

**Affiliations:** ^1^Laboratoire Angevin de Recherche en Ingénierie des Systèmes, EA7315, University of Angers – Université Nantes Angers Le MansAngers, France; ^2^Department of Radiology, Angers University Hospital, University of Angers – Université Nantes Angers Le MansAngers, France; ^3^Department of Neuroscience, Division of Geriatric Medicine and Memory Clinic – Angers University Hospital; UPRES EA 4638 – University of Angers, Université Nantes Angers Le MansAngers, France; ^4^Robarts Research Institute, Department of Medical Biophysics, Schulich School of Medicine and Dentistry, University of Western Ontario, LondonON, Canada; ^5^Laboratoire de Biologie Neuro-Vasculaire et Mitochondriale Intégrée, UMR CNRS 6214 INSERM U1083, University of AngersAngers, France; ^6^Institute of Biomedical Problems, Russian Academy of SciencesMoscow, Russia; ^7^Centre National des Etudes SpatialesParis, France; ^8^Department of Anesthesia and Critical Care, Angers University Hospital – University of Angers, Université Nantes Angers Le MansAngers, France; ^9^Department of Medicine, Division of Geriatric Medicine, Sir Mortimer B. Davis – Jewish General Hospital and Lady Davis Institute for Medical Research, McGill University, MontrealQC, Canada; ^10^Dr. Joseph Kaufmann Chair in Geriatric Medicine, Faculty of Medicine, McGill University, MontrealQC, Canada; ^11^Clinical Research Center, Angers University Hospital, University of Angers – Université Nantes Angers Le MansAngers, France; ^12^Department of Physical and Rehabilitation Medicine, Angers University Hospital, University of Angers – Université Nantes Angers Le MansAngers, France

**Keywords:** locomotion, walking, supplementary motor area, sensorimotor cortex, mental imagery

## Abstract

Human locomotion is a complex sensorimotor behavior whose central control remains difficult to explore using neuroimaging method due to technical constraints, notably the impossibility to walk with a scanner on the head and/or to walk for real inside current scanners. The aim of this functional Magnetic Resonance Imaging (fMRI) study was to analyze interactions between two paradigms to investigate the brain gait control network: (1) mental imagery of gait, and (2) passive mechanical stimulation of the plantar surface of the foot with the Korvit boots. The Korvit stimulator was used through two different modes, namely an organized (“gait like”) sequence and a destructured (chaotic) pattern. Eighteen right-handed young healthy volunteers were recruited (mean age, 27 ± 4.7 years). Mental imagery activated a broad neuronal network including the supplementary motor area-proper (SMA-proper), pre-SMA, the dorsal premotor cortex, ventrolateral prefrontal cortex, anterior insula, and precuneus/superior parietal areas. The mechanical plantar stimulation activated the primary sensorimotor cortex and secondary somatosensory cortex bilaterally. The paradigms generated statistically common areas of activity, notably bilateral SMA-proper and right pre-SMA, highlighting the potential key role of SMA in gait control. There was no difference between the organized and chaotic Korvit sequences, highlighting the difficulty of developing a walking-specific plantar stimulation paradigm. In conclusion, this combined-fMRI paradigm combining mental imagery and gait-like plantar stimulation provides complementary information regarding gait-related brain activity and appears useful for the assessment of high-level gait control.

## Introduction

Gait is a complex motor behavior that consists of rhythmic movements, involving several sensorimotor systems, under the control of a balance between automatic and cognitive controlled processes (Clark, 2015). Interaction between different key areas of the central nervous system, from spinal generators to the cortex, is required to produce gait (Duysens and Van de Crommert, 1998; Rossignol et al., 2006). Multimodal sensory information must be constantly integrated to deal with changes in the environment (Maurer et al., 2000).

The study of cortical and subcortical network activations during real gait is technically very complex due to technical constraints, size and weight of current MRI scanners and because of the impossibility to walk with an MRI on the head and/or to walk for real inside an MRI scanner. It has only been carried out in a few neuroimaging-type studies, mainly using single photon emission computed tomography (SPECT), positron emission tomography (PET), and functional Near-Infrared Spectroscopy (fNIRS). SPECT and PET have been used to explore the brain areas specifically activated after real steady-state locomotion (Fukuyama et al., 1997; Tashiro et al., 2001; Malouin et al., 2003; la Fougère et al., 2010), however, these methods are invasive due to the injection of a radiotracer and the radiation. In contrast fNIRS appears as a promising tool because of the possibility of investigating non-invasively brain activity during real gait (Mirelman et al., 2014; Perrey, 2014; Maidan et al., 2015). In the same way, electroencephalography (EEG) also stands out as an electrophysiological technique allowing to record cortical activity during actual walking (Wagner et al., 2012, 2014; Seeber et al., 2014, 2015). EEG has the advantage of high temporal resolution that is missing in both functional Magnetic Resonance Imaging (fMRI) and fNIRS, wich allows examining neural activity relative to specific gait phases. However, both fNIRS and EEG suffer from depth limitation, poor spatial resolution and difficulties to explore whole brain (Torricelli et al., 2014). The development of fMRI since the 1990s has made the non-invasive imaging of human brain activity during active or passive tasks possible. However, active gait cannot be performed directly in the scanner. It is important to increase understanding of the supraspinal control of gait (i.e., the “brain gait control network”) to improve treatment of gait disorders resulting from brain lesions, including neurodegenerative diseases such as Parkinson’s or Alzheimer’s disease (Annweiler et al., 2013; Peterson et al., 2014b). However, the establishment of an exploratory paradigm of gait using fMRI remains a daunting task (Sahyoun et al., 2004; Mehta et al., 2009; Jaeger et al., 2014).

Indirect paradigms have generally been used to investigate the brain areas associated with gait control. Data are still partial and do not provide a full overview of the “gait control network”. There are three main challenges relating to the fMRI study of gait: (i) the subject must remain supine and keep the head still; (ii) any stimulating equipment should be MR-compatible and generate no artifacts in the magnetic field; (iii) the paradigm should be functionally similar to real gait, including afferent feedback (visual and somatosensory information inherent to gait), and the cognitive load should be similar. This latter point is the most challenging. Current fMRI studies of gait control have focused on either somatosensory or cognitive processing, but not on their interaction. For instance, brain activity during active or passive ankle dorsi- and plantarflexion, a critical component of gait (Dobkin et al., 2004), has been explored using fMRI (Dobkin et al., 2004; Sahyoun et al., 2004; Trinastic et al., 2010), however, this single joint movement is only one part of the complex gait cycle and does not induce the same degree of cognitive load.

Paradigms for the evaluation of cognitive process associated with gait are generally based on action observation and mental imagery of gait (Bakker et al., 2007, 2008; Iseki et al., 2008; Wang et al., 2008b; Deutschländer et al., 2009; Zwergal et al., 2012; Blumen et al., 2014), assuming a functional equivalence between intending, imagining, observing, and performing an action (Grèzes and Decety, 2001; Iseki et al., 2008). Mental imagery (e.g., imagination of walking) is defined as the ability to mentally plan and perform an action (e.g., walking) without overtly performing it (Decety, 1996) and, *a fortiori*, without the sensory inputs that are generated during the action (for example during gait without the pressure of the feet on the soles). fMRI-compatible robotic devices have been developed to evaluate brain activity during multi-joint movements (Mehta et al., 2009; Jaeger et al., 2014), however, their use involves many technical constraints, and the movements produced are more related to cycling than gait.

Some studies have used somatosensory stimulation of the foot, particularly vibrotactile stimulation (Golaszewski et al., 2006) to explore the feedback loops that contribute to the control of standing and walking. An MRI-compatible system of boots, the “Korvit” system (Kremneva et al., 2012), generates well-controlled, reproducible mechanical stimulation of the plantar surface of the foot by the application of pneumatic pressure on the relevant support zones, in a pattern which reproduces the pressures generated during gait. This system has been used to evaluate brain activity during plantar stimulations that mimicked standing and slow gait (Kremneva et al., 2012). However, it is not known if the brain activity generated was the result of the fact that the plantar pressures represented pressures produced during gait, or if it was simply the result of the pressure on the feet. It is necessary to compare an organized (gait-like) sequence of plantar stimulations with a chaotic (non-gait-like) sequence, in order to differentiate brain activity which is specific to proprioception from that which is related to gait. Moreover, an fMRI task relating to the cognitive load of gait was not included, thus common zones of activation between somatosensory processing and cognitive processing associated with gait were not evaluated. It is therefore not clear from the available literature whether brain activity generated by mental imagery of gait is similar to activity generated by gait-like somatosensory stimulation.

We developed a novel combined-paradigm to analyze interactions between activity produced during plantar stimulation with the Korvit system and mental imagery of gait. We hypothesized that this paradigm would provide complementary information regarding gait-related brain activity. This approach may allow more a comprehensive analysis of the neural networks involved in the high-level control of gait. The aims of this study were (i) to evaluate if a combined analysis of two paradigms carried out in a single fMRI session (mental imagery of gait and organized plantar stimulation) would provide more information than separate analyses, and (ii) to compare fMRI patterns of activation between organized (“gait like”) and chaotic sequences of plantar stimulations.

## Materials and Methods

### Healthy Volunteers

Eighteen healthy volunteers (7 women and 11 men), aged from 20 to 40 years (mean age, 27 ± 4.7 years), with no neurological or orthopedic disorders, from our local clinical research center were included. All participants were right-handed (confirmed using the Edinburgh Handedness Inventory for determining the dominant hand) (Oldfield, 1971). In order to test subject’s cognitive integrity a Mini Mental State Examination was performed.

### Ethics Statement

The study was conducted in accordance with the ethical standards of the Helsinki Declaration (1983). Written informed consent was obtained at enrolment and the entire study protocol was approved by the University of Angers Ethical Review Committee (Comité de protection des personnes, CPP ouest II, Angers, France, n° A.C = 2014-A01593-44, n°CPP: 2014/32).

### Korvit Plantar Pressure Simulator

**FIGURE 1:**
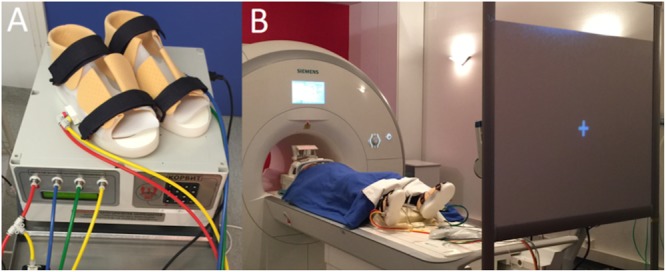
**(A)** Korvit MRI-compatible simulator, composed of a control unit, air ducts and pneumatic boots. **(B)** Participant lying in the scanner and wearing the Korvit boots, looking at a screen through a prism.

The MRI-compatible Korvit simulator (**Figure 1**) was used to mechanically stimulate the plantar support zones of the feet. The Korvit system consists of a pair of plastic boots connected to a compressor by air cables. Three sizes of boots are available. The inflatable rubber chambers were placed under the phalanges and the heel of each participant, and produced a pressure of 40 kPa on these zones. The Korvit simulator was first developed by IBMP (Moscow, Russia) for cosmonauts to simulate walking in space and to reduce neuromuscular impairment following prolonged weightlessness (Layne and Forth, 2008). The device is manufactured by the companies “VIT” (Saint-Petersburg, Russia) and “Center of Aviaspace medicine” (Moscow, Russia).

Two specific modes of stimulation were used in this study – organized and chaotic. The organized mode produced gait-like stimulation of the plantar surfaces of the feet, mimicking a cadence of 120 steps per minute. The cycle was as follows: right heel, right toes, left heel, left toes, and so on. The chaotic mode consisted of a non-gait-like pattern of stimulation: right heel, left toes, left heel, right toes, and so on. The cadence was similar to the organized mode, i.e., 120 “pseudo” steps per minute.

### Magnetic Resonance Imaging Preparation and Data Acquisition

Functional Magnetic Resonance Imaging was performed on a clinical 3T MRI unit (Magnetom Skyra, Siemens, Erlangen, Germany), using a standard transmitter-receiver head coil.

Participants lay comfortably in the scanner, with headphones on to hear the instructions, foam blocks to keep the head still and the Korvit boots on their feet. They looked through a prism at a screen positioned at their feet, facing them. Lights were turned off during image acquisition. The participants were strictly instructed not to move during the entire protocol. Surface EMG was used to monitor activity of the tibialis anterior and soleus muscles to check that participants did not perform any voluntary muscle contractions during the protocol.

A three-dimensional high-resolution T1-weighted volume covering the whole brain was acquired (192 contiguous axial slices, 256 × 256 in-plane matrix, yielding a voxel size of 1 mm × 1 mm × 1 mm) thereby providing an anatomical image for further co-registration and normalization.

An echo planar imaging sequence was used to acquire functional sessions for each participant (repetition time 2280 ms, echo time 30 ms, flip angle 90°, 40 axial slices interleaved, 4.0 mm thick, 0 mm gap, in a 64 × 64 plane matrix, yielding a voxel size of 3.75 mm × 3.75 mm × 4 mm, field of view 240 mm), covering the whole brain, including the cerebellum. Three separate fMRI sessions, including 150 functional volumes per session, were performed for each subject during the same MRI procedure.

### Experimental Design

The fMRI study was organized as a block-design experiment. Each session involved two consecutive conditions. Each condition was performed for 19 s and repeated nine times, for a total session duration of 5 min and 42 s. Three fMRI sessions were carried out for the present analysis.

Session #1 consisted of alternating an ORGANIZED condition and a REST_Organized_ condition. In the ORGANIZED condition, the Korvit boots were activated and produced a structured pattern of pressures, similar to the pattern of foot pressures during gait. The participant was instructed to look at a white cross in the center of a black screen and to remain perfectly still. During the REST_Organized_ condition, the participant continued to look at the cross, but the boots were disabled and no stimulation was applied.

Session #2, composed of a CHAOTIC condition and a REST_Chaotic_ condition, was organized just as session #1, except that Korvit boots were activated with a chaotic pattern which did not mimic foot pressures during gait.

Session #3 consisted of alternating an IMAGINATION condition and a REST_Imagination_ condition. During the IMAGINATION condition, the screen displayed a static picture of an unobstructed forest trail along which the participant had to imagine that he/she was walking. At the beginning of the block, the participant was instructed to “Imagine that you are walking along the trail”. During the REST_Imagination_ condition, the screen displayed an abstract image with the same colors and luminosity as the forest (variations of brown and green), the participant was instructed to “Stop imagining” at the beginning of the block. Each participant received instructions and training for this task before the MRI procedure. After this session, each participant confirmed orally that they performed the imagination task well.

### Image Preprocessing

Functional Magnetic Resonance Imaging data were analyzed using SPM12^1^ (Wellcome Department of Imaging Neuroscience, University College, London, UK) implemented on Matlab (The MathWorks, Natick, MA, USA). First, native space images were corrected for the time delay between different slices (slice timing step). Then, they were realigned to the first volume and unwrapped to correct for head movements and susceptibility distortions. Participants were excluded from analysis if head motion was greater than 3mm or greater than 3° during the whole fMRI session. Coregistration of images from different sessions was achieved using mean echo planar of slice-timed and motion corrected unwrapped images as reference image and 3D T1-weighted anatomical image as source image. The 3D T1 volume was segmented in native-space, using a unified segmentation approach (Ashburner and Friston, 2005). Echo-planar images were rewritten to a final resolution of 3 mm ×3 mm ×3 mm and normalized to the Montreal Neurological Institute template (MNI template) using the forward deformation field generated during segmentation. Finally, functional images were smoothed by an isotropic Gaussian kernel of 8 mm full-width at half-maximum.

### fMRI Statistical Analysis

First level statistical analysis was carried out for each participant by modeling the different conditions as separate regressors in the same general linear model (GLM) (Friston et al., 1995). A high-pass filter with a cut-off of 128 s was used to remove low frequency noise.

Each individual specific design matrix was filled with the following condition order: CHAOTIC, REST_Chaotic_, ORGANIZED, REST_Organized_, IMAGINATION, REST_Imagination_. To reduce artifacts from subject movements, the alignment rigid transformation parameters were also introduced as regressors. Thus, eight contrast images were computed with the following vectors :

(1) CHAOTIC > REST_Chaotic_;
(2) ORGANIZED > REST_Organized_;
(3) (CHAOTIC > REST_Chaotic_) > (ORGANIZED > REST_Organized_);
(4) (ORGANIZED > REST_Organized_) > (CHAOTIC > REST_Chaotic_);
(5) IMAGINATION > REST_Imagination_;
(6) (IMAGINATION > REST_Imagination_) > (ORGANIZED > REST_Organized_);
(7) (ORGANIZED > REST_Organized_) > (IMAGINATION > REST_Imagination_);
(8) ORGANIZED + IMAGINATION > REST_Organized_ + REST_Imagination_.

Then, to make broader inferences about the general population from which the subjects were drawn, each subject’s contrast images from the first level analysis were entered into a random effects second level analysis using one sample *t*-tests. The contrasts 1, 2, 3, and 4 analyzed similarities and differences between brain networks involved in processing organized or chaotic mechanical plantar stimulation. Contrast 5 analyzed the brain network involved in mental imagery of locomotion. Contrasts 6 and 7 analyzed differences between mental imagery and mechanical stimulation, and contrast 8 analyzed interactions between these two tasks.

A threshold of *p* < 0.05, corrected for multiple comparisons based on the false discovery rate (FDR), was applied to the resulting statistical parametric maps. Only clusters with a minimum extent of ten contiguous voxels are reported. Anatomical correlates of clusters of activation were determined visually and with the help of probabilistic cytoarchitectonic maps implemented in the Anatomy toolbox (Eickhoff et al., 2005).

## Results

Data from one participant were excluded from the analysis due to excessive head motion during fMRI acquisition (12 mm and 9°). The results from all other participants (*n* = 17) are presented below.

### Brain Activation during ORGANIZED and CHAOTIC Korvit Plantar Stimulation

**FIGURE 2:**
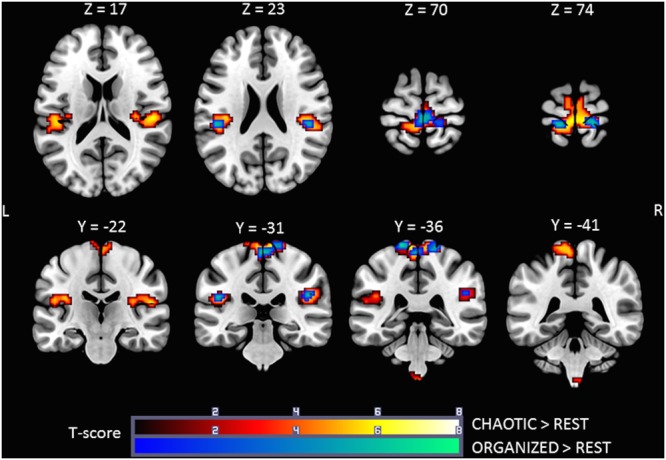
**Statistical parametric maps for the CHAOTIC > REST (warm colors) and ORGANIZED > REST (cold colors) contrasts, *p*-value threshold of <0.05 FDR-corrected at voxel level with a minimum cluster extent of 10 contiguous voxels; images are presented according to neurological convention (R = right, L = left)**. See text for details.

Brain activity revealed by the ORGANIZED > REST and CHAOTIC > REST contrasts is presented in **Figure 2** and represents the networks involved in processing afferent information from mechanical foot pressure. The biggest cluster was centered on the right (R) and left (L) paracentral lobules, with lateral extensions on the post central giri (R and L), a posterior extension on the precuneus (L), and a small anterior extension to the Supplementary Motor Areas (SMA) (R and L). Most of this cluster can be functionally assimilated to the primary sensorimotor cortex (SM1, Brodmann areas = BA 1, 2, 3, and 4), particularly to the mesial parts, which are specific to the lower limbs. Two other clusters were symmetrically centered on the rolandic operculum with significant extensions to the superior temporal giri posteriorly and supramarginal giri superiorly, and may be functionally assimilated to the secondary somatosensory cortex (S2) (Eickhoff et al., 2010). Of note, the brain activations in the ORGANIZED and CHAOTIC conditions was anatomically similar, with an almost complete overlap. Details on location are given in **Table 1**.

**Table 1:** Significant clusters and their corresponding activation peaks for the ***CHAOTIC > REST*** and ***ORGANIZED > REST*** contrasts.

Cluster #	Voxels	Anatomical region		MNI coordinates (mm)			*T*-score
				*x*	*y*	*z*	
***CHAOTIC > REST***							
1	280	Postcentral Gyrus (S1)	R	15	–31	77	6.72
		Postcentral Gyrus (S1)	L	–18	–37	77	6.70
		Paracentral Lobule (SM1)	L	–6	–37	68	6.39
		Paracentral Lobule (SM1)	R	6	–31	71	6.33
2	128	Superior Temporal Gyrus (S2)	R	51	–25	17	6.15
		Rolandic Operculum (S2)	R	39	–25	23	5.79
		SupraMarginal Gyrus (S2)	R	48	–31	26	5.78
		Insula Lobe	R	33	–22	20	5.05
3	93	Insula Lobe	L	–36	–22	20	5.89
		Rolandic Operculum (S2)	L	–42	–31	20	5.83
***ORGANIZED > REST***							
1	55	Paracentral Lobule (SM1)	L	–3	–34	68	6.12
		Postcentral Gyrus (S1)	R	18	–34	74	6.10
2	13	Postcentral Gyrus (S1)	L	–18	–34	77	7.51
3	13	Rolandic Operculum (S2)	R	45	–28	23	5.55
		SupraMarginal Gyrus	R	48	–31	26	5.42
4	12	SupraMarginal Gyrus	L	–48	–31	23	5.91

*Voxel level *p*-value < 0.05 FDR corrected, cluster-size threshold 10 voxels; *x,y,z* = the original SPM coordinates in millimeters of the MNI space; in case of multiple peaks in the same anatomic area of a cluster, only the maximal peak is reported; SM1 = primary sensorimotor cortex; S1 = primary somatosensory cortex; S2 = secondary somatosensory cortex.*

### Comparison of Brain Activation during ORGANIZED and CHAOTIC Stimulations

The (ORGANIZED > REST) > (CHAOTIC > REST) and (CHAOTIC > REST) > (ORGANIZED > REST) contrasts did not reveal any significant activation. No difference in activation was found between organized, gait-like mechanical plantar stimulation and the non-organized, chaotic pattern.

### Brain Activation during Gait IMAGINATION Task

**FIGURE 3:**
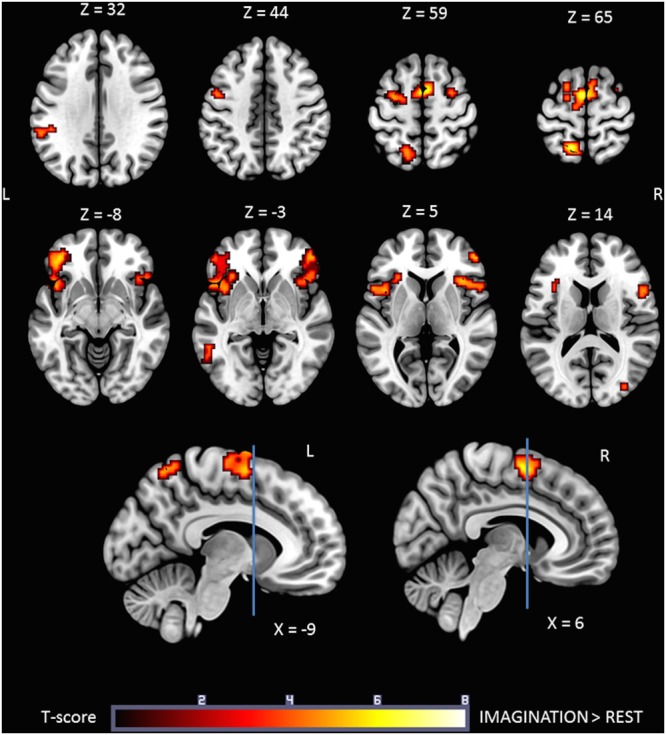
**Statistical parametric map for the IMAGINATION > REST contrast *p*-value threshold of <0.05 FDR-corrected at voxel level with a minimum cluster extent of ten contiguous voxels; images are presented according to neurological convention (R = right, L = left).** Blue lines in the sagittal images represent the vertical plane passing through the anterior commissure, separating pre-SMA (anteriorly) and SMA proper (posteriorly). See text for details.

**Figure 3** shows the statistical parametric map for the IMAGINATION > REST contrast, which represents brain activity during the imagined gait task. Bilateral clusters in the frontal inferior gyrus and anterior insula were dominant. The foremost part of these clusters relates to the ventrolateral prefrontal cortex (BA 44, 45, and 47). Active voxels were also located in motor-related areas such as SMA (R and L; BA = 6) and the dorsal premotor cortex (R and L, BA = 6). More precisely, activations concerned the SMA-proper (R and L) and the pre-SMA (R). Finally, additional clusters were found in the Middle Temporal Gyrus (L), Middle Occipital Gyrus (R), SupraMarginal Gyrus (L) and Precuneus/Parietal Sup (L). Details regarding location are provided in **Table 2**.

**Table 2:** Significant clusters and their corresponding activation peaks for the ***IMAGINATION > REST*** contrast.

Cluster #	Voxels	Anatomical region		MNI coordinates (mm)			*T*-score
				*x*	*y*	*z*	
1	311	SMA-proper + pre-SMA	R	3	–1	62	6.98
		SMA-proper	L	–3	–4	65	6.67
		Precentral Gyrus	L	–30	–4	56	5.44
		Superior Frontal Gyrus	L	–18	8	71	5.23
		Paracentral Lobule	L	–12	–13	71	5.11
2	311	Middle Orbital Gyrus	L	–39	44	–7	6.00
		IFG (p. Orbitalis)	L	–42	38	–7	5.87
		Insula Lobe	L	–42	14	–10	5.30
		IFG (p. Triangularis)	L	–51	17	–1	5.16
3	238	Middle Frontal Gyrus	R	48	44	2	7.42
		IFG (p. Opercularis)	R	54	14	11	5.54
		Insula Lobe	R	36	17	8	5.51
		IFG (p. Triangularis)	R	48	26	–1	4.34
4	85	Precuneus	L	–15	–55	65	7.10
5	29	Middle Frontal Gyrus	R	30	–1	56	4.40
6	24	SupraMarginal Gyrus	L	–60	–37	29	5.12
7	23	Precentral Gyrus	L	–45	–4	47	5.09
8	23	Middle Occipital Gyrus	R	39	–82	23	4.91
9	21	Middle Temporal Gyrus	L	–54	–46	–4	4.65

*Voxel level *p*-value < 0.05 FDR corrected, cluster-size threshold 10 voxels; *x,y,z* = the original SPM coordinates in millimeters of the MNI space; in case of multiple peaks in the same anatomic area of a cluster, only the maximal peak is reported; SMA = Supplementary Motor Area; IFG = Inferior Frontal Gyrus.*

### Comparison of Brain Activation during ORGANIZED and IMAGINATION Conditions

**FIGURE 4:**
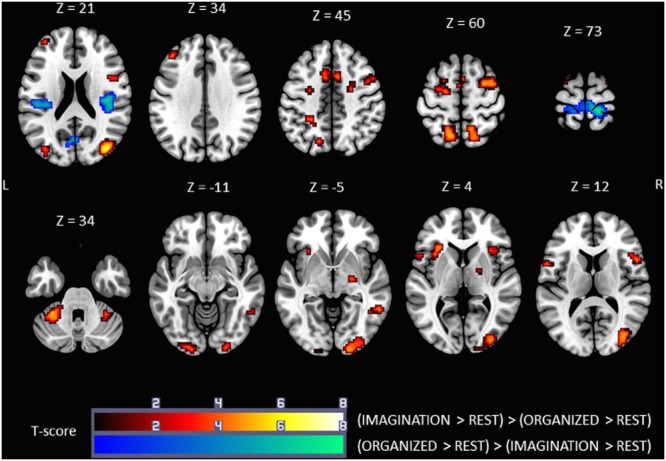
**Statistical parametric maps for the (IMAGINATION > REST) > (ORGANIZED > REST) (warm colors) and (ORGANIZED > REST) > (IMAGINATION > REST) (cold colors) contrasts, representing differences in brain activity between gait-like plantar stimulation and mental imagery of gait, *p*-value threshold of <0.05 FDR-corrected at voxel level with a minimum cluster extent of ten contiguous voxels; images are presented according to neurological convention (R = right, L = left).** See text for details.

Comparison of the ORGANIZED condition to the IMAGINATION condition with the (ORGANIZED > REST) > (IMAGINATION > REST) contrast (**Figure 4**, cold colors) showed similar activations to the contrast ORGANIZED > REST in the primary sensorimotor cortex (SM1; R and L), especially areas relating to the lower limb, and rolandic operculum (S2; R and L). Note that an additional cluster was found in the Left Calcarine Gyrus.

Comparison of the ORGANIZED condition with the IMAGINATION condition using the (IMAGINATION > REST) > (ORGANIZED > REST) contrast (**Figure 4**, warm colors) showed significant clusters in SMA (R and L), the dorsal premotor cortex (R and L), the precuneus/superior parietal area (R and L) and anterior insula (R and L). Activity in the prefrontal cortex was much less extensive. This pattern of activation appears very similar to the brain network found in the IMAGINATION condition. Other zones of activity, not found in the IMAGINATION condition, were found in the cerebellum (IV, V, and VI; R and L), the midbrain (R), the visual cortical areas (BA 17, 18, and 19; R and L), the middle cingulum cortex (R and L) and the middle/inferior temporal gyrus (R). Details on location are given in **Tables 3**, **4**.

**Table 3:** Significant clusters and their corresponding activation peaks for the ***(IMAGINATION > REST) > (ORGANIZED > REST)*** contrast.

Cluster #	Voxels	Anatomical region		MNI coordinates (mm)			*T*-score
				*x*	*y*	*z*	
1	251	Middle Occipital Gyrus	R	42	–79	17	6.78
		Calcarine Gyrus	R	18	–100	–4	5.85
		Lingual Gyrus	R	21	–97	–10	5.04
2	128	Precentral Gyrus	R	48	8	50	6.14
		Superior Frontal Gyrus	R	33	2	65	5.97
		Middle Frontal Gyrus	R	42	2	59	5.62
3	113	Cerebelum (IV–V)	L	–18	–43	–22	5.82
		Cerebelum (VI)	L	–33	–43	–34	5.82
4	111	Precuneus	L	–15	–55	65	6.72
		Superior Parietal Lobule	L	–15	–70	41	5.01
5	63	Precuneus	R	12	–55	59	4.89
		Superior Parietal Lobule	R	18	–67	59	4.84
6	54	Middle Frontal Gyrus	L	–27	–7	50	4.57
7	53	IFG (p. Opercularis)	R	54	14	11	4.59
		Insula Lobe	R	39	20	8	4.53
8	39	Insula Lobe	L	–30	23	5	4.92
9	29	Middle Temporal Gyrus	R	57	–49	–4	4.57
		Inferior Temporal Gyrus	R	48	–52	–7	4.39
10	28	Middle Frontal Gyrus	L	–42	38	32	5.01
11	27	Lingual Gyrus	L	–18	–97	–13	4.96
12	21	Middle Cingulate Cortex	L	–9	14	44	4.47
13	20	Superior Frontal Gyrus	L	–21	5	65	5.36
14	18	Middle Occipital Gyrus	L	–36	–82	17	4.35
15	16	IFG (p. Opercularis)	L	–54	14	5	4.36
16	15	Middle Cingulate Cortex	R	9	11	44	4.40
17	14	Cerebelum (Crus 1)	R	39	–46	–34	4.50
18	13	SMA-proper	R	3	5	59	4.16
		SMA-proper	L	–3	–1	59	4.09

*Voxel level *p*-value < 0.05 FDR corrected for a cluster-size threshold of 10 voxels; *x,y,z* = the original SPM coordinates in millimeters of the MNI space; in case of multiple peaks in the same anatomic area of a cluster, only the maximal peak is reported; SMA = Supplementary Motor Area; IFG = Inferior Frontal Gyrus.*

**Table 4:** Significant clusters and their corresponding activation peaks for the contrast ***(ORGANIZED > REST) > (IMAGINATION > REST)*.**

Cluster #	Voxels	Anatomical region		MNI coordinates (mm)			*T*-score
				*x*	*y*	*z*	
1	133	Postcentral Gyrus (S1)	R	18	–34	74	7.83
		Paracentral Lobule (SM1)	R	6	–28	68	5.88
		Paracentral Lobule (SM1)	L	–3	–31	68	5.35
2	71	Rolandic Operculum (S2)	R	42	–19	20	6.60
		Insula Lobe	R	36	–22	23	6.53
3	36	Rolandic Operculum (S2)	L	–45	–25	20	5.54
		Insula Lobe	L	–36	–25	23	5.22
4	25	Postcentral Gyrus (S1)	L	–18	–34	77	6.62
5	22	Calcarine Gyrus	L	–3	–70	20	4.99

*Voxel level *p*-value < 0.05 FDR corrected, cluster-size threshold 10 voxels; *x,y,z* = the original SPM coordinates in millimeters of the MNI space; in case of multiple peaks in the same anatomic area of a cluster, only the maximal peak is reported; SM1 = primary sensorimotor cortex; S1 = primary somatosensory cortex; S2 = secondary somatosensory cortex.*

### Brain Activations Common to the ORGANIZED and IMAGINATION Conditions

**FIGURE 5:**
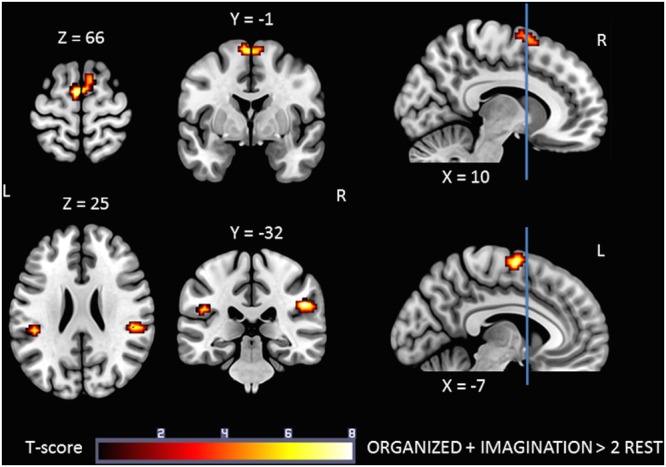
**Statistical parametric map for the ORGANIZED + IMAGINATION > REST_Organized_ + REST_Imagination_ contrast representing common activations between gait-like plantar stimulation and mental imagery of walking; *p*-value threshold of <0.05 FDR-corrected at voxel level with a minimum cluster extent of ten contiguous voxels; images are presented according to neurological convention (R = right, L = left).** Blue lines in the sagittal images represent the vertical plane passing through the anterior commissure separating pre-SMA (anteriorly) and SMA proper (posteriorly). See text for details.

The IMAGINATION + ORGANIZED > REST_Imagination_ + REST_Organized_ contrast was designed to highlight common statistical activity between the two main conditions: ORGANIZED and IMAGINATION. The statistical parametric map related to this contrast (**Figure 5**) revealed significant bilateral clusters in the secondary somatosensory cortex (at the intersection of the rolandic operculum, temporal superior gyrus and the supramarginal gyrus) and the SMA. More specifically, the SMA-proper was bilateral activated and the pre-SMA was only activated on the right side (Picard and Strick, 1996) (See sagittal slices in **Figure 5**). Details on location are given in **Table 5**.

**Table 5:** Significant clusters and their corresponding activation peaks for the ***IMAGINATION + ORGANIZED > 2 REST*** contrast.

Cluster #	Voxels	Anatomic region		MNI coordinates (mm)			*T*-score
				*x*	*y*	*z*	
1	52	SMA-proper	L	–6	–4	65	8.38
		SMA-proper + pre-SMA	R	9	8	65	5.92
2	30	SupraMarginal Gyrus (S2)	R	51	–31	29	11.04
3	14	Superior Temporal Gyrus (S2)	L	–45	–34	23	7.06

*Voxel level *p*-value < 0.05 FDR corrected for cluster-size threshold of 10 voxels; *x,y,z* = the original SPM coordinates in millimeters of the MNI space; in case of multiple peaks in the same anatomic area of a cluster, only the maximal peak is reported; SMA = Supplementary Motor Area; S2 = secondary somatosensory cortex.*

## Discussion

This fMRI study was designed to analyze gait-related patterns of brain activation. Two different paradigms described in the literature were combined for the first time: (1) mechanical plantar stimulation using the Korvit system, and (2) mental imagery of gait. The neural networks activated during these two conditions differed, however, some activation was common to both, notably in the bilateral SMA-proper, right pre-SMA and bilateral S2, thus emphasizing a potential key role of the SMA in gait control.

### Brain Activity during Mechanical Plantar Stimulation using the Korvit Boots

Both organized and chaotic plantar stimulation using the Korvit system activated SM1 and S2 bilaterally. The Korvit boots were designed to stimulate the supporting areas of the feet, which have a maximal density of mechanoreceptors, in order produce gait-like somatosensory inflow (Kremneva et al., 2012). Activation of SM1 was principally in the paracentral lobule, particularly on the medial surface, which relates to the lower limbs. No consistent EMG activity was recorded during the MRI procedure, thus the activation of M1 during the passive plantar stimulation was not related to muscle contractions. This finding is consistent with the assumption that motor areas may contribute to both sensory processing and motor output (Naito et al., 2007). Activation of S2 was located in the rolandic operculum areas, at the depth of the Sylvian fissure. S2 is considered to be an associative somatosensory area, which performs high-order functions such as the integration of stimuli and memory from both sides of the body (Chen et al., 2008). SM1 and S2 were not activated in the IMAGINATION condition, suggesting that mechanical plantar stimulation is a useful paradigm, providing additional “natural” stimulation of the plantar surface of the foot, which is a key component of gait. Activation of these areas was also found in previous reports using various plantar stimulators (vibrotactile, pneumatic etc.) (Golaszewski et al., 2006; Kremneva et al., 2012; Hao et al., 2013). Moreover, the ORGANIZED > IMAGINATION contrast revealed activity in SM1 and S2 and, interestingly, was similar to the results of the motor study by Gerardin et al. (Gerardin et al., 2000), which demonstrated greater activation of both these regions during movement execution compared to mental imagery. This finding supports the use of plantar stimulation, in addition to mental imagery of gait, to assess the “direct locomotor pathway”, a pathway which involves sensori-motor areas that are activated to a greater extent during real gait (Hamacher et al., 2015). Since it is currently not feasible to acquire BOLD signals during real gait, our combined paradigm may be a useful substitute.

There was no difference between the ORGANIZED and CHAOTIC conditions. Two aspects of these conditions were identical: the cadence was set to 120 steps per minute, and the force of the mechanical stimuli was constant. Only the stimulation sequence of the different support zones of the soles was different, with a “gait like” pattern and a de-structured pattern, respectively. No suprathreshold clusters were found in the ORGANIZED > CHAOTIC or CHAOTIC > ORGANIZED contrasts, suggesting the possible need to improve our plantar stimulation paradigm. Two main hypotheses could explain such a result: (1) the stimulation pattern was gait-like and not real gait *per se*, (2) the stimulation pattern could suffer from a lack of power. Further studies involving new foot pressure paradigms are necessary to explore these hypotheses.

### Mental Imagery of Gait

The IMAGINATION condition activated a broad neuronal network, in particular the SMA-proper, pre-SMA, dorsal premotor cortex, ventrolateral prefrontal cortex, anterior insula, and precuneus/superior parietal areas. These findings are consistent with numerous studies of locomotor imagery (Jahn et al., 2004, 2008; Sacco et al., 2006; Wagner et al., 2008; Wang et al., 2008a,b; la Fougère et al., 2010; van der Meulen et al., 2014), including a recent systematic review by Hamacher et al. (2015). However, we did not find significant activations of the cingulate cortex, basal ganglia, parahippocampal gyrus, mesencephalic locomotor region or cerebellum, which have been reported in other studies (Jahn et al., 2004, 2008; Sacco et al., 2006; Wang et al., 2008b; la Fougère et al., 2010; van der Meulen et al., 2014; Peterson et al., 2014b). This difference may be related to the choice of the control condition used (REST), in which the participant was instructed to stop the imagination task, while in most mental imagery studies, subjects were instructed to imagine standing or lying as the control condition. Furthermore, the mental imagery task in the present study may have been less complex than the locomotion imagery tasks used in other studies, which included goals, obstacles or dual tasks. Some brain areas such as the anterior cingulate cortex and the prefrontal cortex are particularly active in more controlled gait (Hamacher et al., 2015). Nevertheless, the (IMAGINATION > REST) > (ORGANIZED > REST) contrast, which provides greater statistical power, revealed significant activations in the cingulate cortex, midbrain and cerebellum.

It is not surprising that activity was found in the SMA-proper since this area is crucial for learned, self-initiated, voluntary motor behavior, especially relating to the initiation of a task (Burton et al., 1996; Freund, 1996; Koenraadt et al., 2014). Moreover, the SMA-proper is involved in anticipating and correcting posture during motor tasks, such as the coordination of the lower extremities (Brinkman, 1984; Nakagawa et al., 2016). Pre-SMA plays a role in high-level planning, such as sequencing and preparing complex tasks, particularly internally generated, visually guided tasks. Pre-SMA is mainly connected to prefrontal areas, whereas the SMA-proper is linked to the primary motor areas (Picard and Strick, 1996). The results showed no activation of M1, consistently with previous reports of reduced activation of M1 during imagined movements compared to real movements (Schnitzler et al., 1997; Gerardin et al., 2000; la Fougère et al., 2010).

Activity in the posterior parietal cortex was only observed in the left hemisphere, consistent with the fact that all subjects were right-handed (Gerardin et al., 2000). It is well known that lesions of the left parietal lobe can produce bilateral apraxia, without any sensory-motor impairment (De Renzi et al., 1982; Heilman et al., 1982), and more severe deficits of mental imagery of complex movements (Sirigu et al., 1995). This left-hemisphere dominance was associated with a bigger cluster of activation in the left SMA-proper, and may be related to the potent connections between superior parietal areas and SMA-proper.

Interestingly, activity in pre-SMA was also lateralized, with significant voxel activation only on the right side, and more particularly in the caudal part of the pre-SMA. A previous study of connectivity found a left-to-right and rostro-caudal gradient of increasing connectivity with attentional networks within the pre-SMA (Ter Minassian et al., 2014). There was also a negative correlation between the intrinsic connectivity network of the right caudal pre-SMA and the default mode network. These results demonstrate the complexity of locomotor tasks, which require a greater focus on the external environment.

Compared with studies that involved simple, single-joint active movements of the lower limbs, such as ankle plantar/dorsiflexion (Dobkin et al., 2004; Sahyoun et al., 2004; Ciccarelli et al., 2005), the mental imagery task in the present study evoked significant additional activity in the dorsal premotor cortex and the posterior parietal cortex. The conjunction of activity in the latter two areas during mental imagery of gait is consistent with neurophysiological studies in monkeys, showing highly specific reciprocal connections between the parietal and frontal cortices (Chafee and Goldman-Rakic, 1998, 2000; Matelli et al., 1998). Similar activations have also been found during imagery of hand movements. The pre-motor - parietal network is a functional loop for movement ideation, planning and locomotor representation (Gerardin et al., 2000). Furthermore, premotor activity is known to be modulated by the prefrontal associative cortex, in particular the orbitofrontal cortex, which supports motivational aspects of behavior (Carmichael and Price, 1995; Rolls, 2000). In the present study, activity in these areas was highly significant in the IMAGINATION condition.

### Activity Common to Plantar Stimulation and Motor Imagery of Gait

To our knowledge, this is the first study to have identified common brain areas activated during plantar stimulation and motor imagery of gait. The statistical parametric maps corresponding to the IMAGINATION + ORGANIZED > 2 REST contrast showed significant clusters in SMA-proper bilaterally, right pre-SMA, and S2 bilaterally (**Figure 5**). This suggests that these are key areas for the control of gait.

Studies in monkeys have demonstrated the essential role of the SMA-proper in the regulation of locomotion, particularly complex locomotion such as climbing or leaping, supporting the hypothesis that SMA-proper is very active in the control of postural stability during stance and walking, in the coordination of temporal sequences of movements and in planning internally generated movements (Penfield and Welch, 1951; Mushiake et al., 1990; Shima and Tanji, 1998; Graziano et al., 2005; Graziano and Aflalo, 2007). Similarly, lesions of the SMA-proper in humans cause impaired execution of sequential movements (Gaymard et al., 1990; Ryu et al., 2013). In patients with Parkinson’s disease, SMA activity increases with the complexity of the locomotor task (Peterson et al., 2014a). Moreover, SMA activity and gait velocity have been found to be correlated (Harada et al., 2009).

SMA thus appears to be highly involved in the control of the human gait cycle. SMA acts as an intermediary between somatosensory information and motor results. SMA-proper receives inputs from the primary somatosensory cortex and the parietal cortex (BA 5) and has projections to the primary motor cortex and the spinal cord (Inase et al., 1999; Johansen-Berg et al., 2004; Behrens et al., 2006). Thus, the activation of SMA during both the mental imagery task and the passive plantar stimulation further supports the key role of SMA in complex sequential motor behaviors such as locomotion. It is of interest that EEG studies have recently highlighted the involvement of the parietal cortex in visuomotor adaptation during walking and the involvement of the prefrontal cortex (notably the SMA) in gait adaptation (Wagner et al., 2014, 2016). These results are consistent with our present fMRI findings.

Activation of the right pre-SMA probably relates to the strong attentional component of both conditions tested (IMAGINATION and ORGANIZED), compared to the REST conditions, in which the participant was engaged in internal thoughts.

Finally, the significant clusters found bilaterally in S2 provide further evidence that somatosensory feedback from the sole of the foot is critical for gait control.

### Limitations of the Study

The use of a combined-paradigm to explore gait, associating plantar somatosensory feedback with an imagination task, was, of course, not as ideal as real gait, however, in view of the current constraints relating to the use of MRI, we consider that it is an appropriate, non-invasive solution, able to explore the whole brain.

An important limitation of this study was the likely significant variations in the achievement of the imagery task by the participants. Although each subject confirmed orally that they performed the imagination task well, it is well known that imagery ability varies widely across individuals (van der Meulen et al., 2014). Furthermore, specific imagery tasks are required to distinguish differences in brain activation according to the various components of gait (gait initiation, steady-state, termination, velocity, etc.).

Moreover, as we tried to explore plantar somatosensory feedback, paradigms could be improved to explore other inputs, like those concerning the muscle spindles of the lower extremities or the vestibular system.

Finally, we fixed the step rate at 120 steps per minute for our gait-like plantar stimulation pattern in order to standardize activations. However future analyses of the influence of participant’s proper cadence on cerebral activity will be of interest.

## Conclusion

To our knowledge, this is the first study to combine plantar stimulation and mental imagery of gait, in the same participants, during a single fMRI procedure, thus allowing the assessment of several dimensions of high-level gait control. The results confirmed that mental imagery of gait is useful, and that the somatosensory feedback loops generated from the feet during gait may be assessed using bilateral plantar mechanical stimuli. We further showed that there was no difference in the pattern of brain activation between organized and chaotic patterns of stimulation. This result does not support the existence of a particular zone in the brain for the integration of the plantar sensory sequence own to gait. Finally, we found common patterns of activation between mental imagery and gait-like plantar stimulation, specifically in SMA-proper bilaterally and right pre-SMA. This emphasizes the potential key role of SMA in gait control, acting as a relay for sensory inputs and complex motor outputs.

## Author Contributions

The ‘IRMarche’ was conceived by MD. The present analysis was conceived and performed by ML under the supervision of MD. All authors contributed to gathering the data, and interpretation of the data. All authors revised and critically appraised the intellectual content of the manuscript, and approved the final version.

## Conflict of Interest Statement

The authors declare that the research was conducted in the absence of any commercial or financial relationships that could be construed as a potential conflict of interest.

## Footnotes

1 http://www.fil.ion.ucl.ac.uk/spm/
